# Geosocial Tools for Community Diagnosis and Health Promotion

**DOI:** 10.1177/10901981241294230

**Published:** 2024-11-06

**Authors:** Yang Li, Dario Spini, Cecilia Delgado Villanueva

**Affiliations:** 1 Institute of Psychology, Swiss Centre of Expertise in Life Course Research, University of Lausanne, Lausanne, Switzerland

**Keywords:** community-based participatory research, geosocial embeddedness, territory, social determinants of health, intersectionality

## Abstract

Growing research highlights the role of local community contexts in health and well-being. Communities function as central arenas for health promotion as individuals and community spaces interact in daily life. To better communicate the linkages between community and health, we demonstrate the use of a set of geosocial tools for community diagnosis and health promotion, conceptually grounded in the framework of geosocial embeddedness, using data from community surveys and participatory research conducted with local inhabitants to engage their competency. Through a web-based interactive map generated using a geographic information system, we identified public territories in the municipality where greater policy attention is needed to enhance health. Through an intersectional matrix produced using multilevel analysis, we identified precise social groups by intersectional categories that are most at risk of loneliness for targeted intervention. A health radar chart on multidomain indicators illustrated information on group dynamics and longitudinal comparisons for health promotion. Overall, these tools offered not only diagnoses of the most vulnerable social groups for tailored interventions but also insight for policymakers to improve infrastructure and material resources to promote health. We conclude that geosocial tools offer a promising approach toward sharpening health communication and improving health in the community.

Communities function as central arenas for health promotion as individuals and community spaces interact in daily life (World Health Organization [WHO], 2017). Both material and social resources in the community contribute to the health and quality of life of local inhabitants (Frumkin, 2003; Gidlow et al., 2010; WHO, 2012). While material resources such as green spaces, pavements, parks, and recreational facilities enhance health through engagement in physical activity and an active lifestyle (Li, 2022; Li & Spini, 2023; Sallis et al., 2012), social resources such as libraries, churches, shops, and café terraces benefit health through social interactions and relationships (Klinenberg, 2018).

Indeed, communities are not simply defined by geographic boundaries in the form of street quarters or districts, as individuals within communities form social bonds and interpersonal ties, establishing boundaries that may be different from geographic delineations (Eng & Blanchard, 2006; Li & Spini, 2022). In this sense, communities are characterized by the social cohesion that develops between people, and the social networks and groups that are present as collective norms, roles, and behaviors (Vacchiano & Spini, 2021). These social groupings may exert influence on communities through social, cultural, and political pathways that in turn impact health (Eng & Blanchard, 2006). Research shows, for instance, that bonding social capital and aggregate social trust impact people’s health at the neighborhood level (Poortinga, 2006). That is, members of the community are embedded in the community context, which includes not only the sum of individuals but also the interpersonal relationships and social groupings that are part of the community and that interact with the community (Brunner & Marmot, 2006; Granovetter, 1985).

## Geosocial Embeddedness and Health Promotion

**Figure 1.**
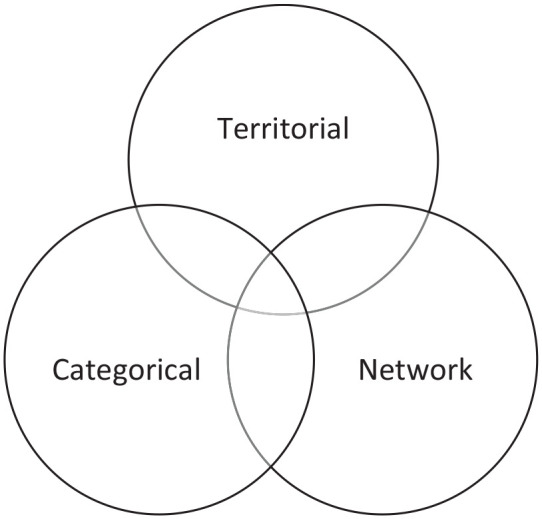
Conceptual Framework of Geosocial Embeddedness. *Source.* Spini (2024) and Luo and MacEachren (2014).

The integrated nature of the geographic and social contexts of communities can be explained by the conceptualization of geosocial embeddedness (Figure 1), a theory framework to help understand the behaviors of social actors in various community contexts: territorial (e.g., regions, places), networks (e.g., social relationships), and social categories (e.g., age, gender, race, and class) (Luo & MacEachren, 2014; Spini, 2024). This multidomain framework joins geographic and social sciences by highlighting the interaction between social processes, social actors, and the role of places (Hess, 2004; Luo & MacEachren, 2014; Spini, 2024). The geosocial framework emphasizes that the three types of embeddedness are not mutually exclusive but interact with one another and may even overlap in the day-to-day activities of the community. On the one hand, street quarters provide the geographic context for inhabitants residing in them, the social relationships and networks of inhabitants give meaning and livelihood to them with a shared sense of the common good. On the other hand, social relationships and networks may reshape neighborhoods over time through wider social processes (Eng & Blanchard, 2006). Policies and interventions that incorporate geosocial embeddedness are key for health promotion as they jointly contribute to a range of health behaviors and outcomes, including physical activity, social isolation, and mental and physical health (Li, 2022; Li et al., 2023; Li & Spini, 2022, 2023).

Understanding the geosocial embeddedness of the community context requires tools that communicate the social relationships and interactions of a community to a broader audience. An important step is to expand from analyzing individuals to understanding social groups to formulate customized interventions (Eng & Blanchard, 2006). A few example questions are worth noting. What tools can help researchers identify groups that are most at risk of social isolation to sharpen intervention targets? How can researchers better identify community spaces that impact inhabitants’ multidomain health, based on the inhabitants’ knowledge of their living spaces? How can researchers better communicate group dynamics in health in the community over time for more effective policies to a nonacademic audience?

Yet, limited tools exist to communicate the geosocial linkage between the community context and health to the public audience, including not only the inhabitants themselves but also the administrators, policymakers, and urban planners. In the spatial sciences, a range of tools have been deployed to communicate research findings, but they largely focused on the geographic distribution of health and the relationship between physical spaces and health outcomes (Luo & MacEachren, 2014), paying scant attention to the social processes or networks inherent in the community, limiting our understanding of the geosocial links in the community and relevant mechanisms to promote health. In the social sciences, existing tools emphasize the role of social identities (gender, age, or race/ethnicity) or social determinants of health. These tools focus on the additive effect of social identities or social factors, and their moderating or mediating effects on health, largely neglecting the multiplicative nature of social factors and identities, or the simultaneous role of the place, thus limiting the development of precise and synergistic interventions for health promotion.

## Geosocial Embeddedness and Community Diagnosis

Community diagnosis contributes to effective policies and interventions that address the realistic needs of the community and its inhabitants. Such diagnosis considers the collective competence of the communities for health promotion. A diagnosis framework that takes into account both the geographic and social aspects of the community was developed by scholars at the University of North Carolina, Chapel Hill (Eng & Blanchard, 2006), with three principles: (1) an extension of the unit of analysis beyond individuals to include social groups and social relationships that collectively impact health outcomes in the community; (2) to uncover existing community conditions, skills, and resources, the diagnosis includes an assessment of the daily life affiliations which are part of the community; (3) the methods of data collection and interpretation are interwoven such that community inhabitants are engaged in defining problems, generating priorities, and observing possible causes and consequences. This community diagnosis framework treats the community as a living laboratory where researchers partner with residents to precisely assess the interactions between territories, social groups, and networks, and how their functions may promote or impede health in the community for targeted health communication.

The geosocial perspective is paramount to community diagnosis as it encompasses not only the territories where social processes take place but also the interactions between territorial, network, and categorical embeddedness, three components of the geosocial framework (Spini, 2024). The geosocial perspective, when applied to community diagnosis, helps to capture experiences with the social, natural, and material dimensions of communities as they jointly determine health outcomes. For example, a participatory community diagnosis with both geographic and social dimensions has led to a proposal for action relating to physical activity promotion among women in a rural community in Basque Country, Spain (Alberdi-Erice et al., 2021), where participants requested greater municipal spaces for physical exercises. Indeed, public spaces in the community embody the geosocial perspective where the territorial and social dimensions integrate to shape health and quality of life.

## The Present Study

To harness the *synergistic* power of geosocial embeddedness, we demonstrate the use of a set of communication tools for community diagnosis and health promotion, based on surveys and participatory research conducted with local inhabitants to engage their knowledge of the environment. These tools facilitate community diagnosis by illuminating the “collective dynamics and functions of relationships within a community as well as interactions between a community and the wider society that can impede or promote” health of its members (Eng & Blanchard, 2006, p. 142). Specifically, we demonstrate three tools on the spatial and social aspects of the community context in relation to health. First, through a web-based interactive map generated using a geographic information system, we identify public spaces in the municipality where greater policy attention is needed to enhance health. Second, through an intersectionality matrix produced using multilevel analysis, we establish precise social groups by intersectional identities that are most at risk of loneliness for targeted intervention. Third, with a health radar chart on multidomain health indicators, we illustrate group dynamics with longitudinal comparisons for health promotion. The overarching objective of these tools is to communicate the links between the geosocial aspects of the community and health to a public audience to aid policy and intervention development.

## Method

### Data and Participatory Research

Data were collected as part of the participatory research project “Cause Commune” (i.e., “common cause,” or “chat in the community” in English) from community-dwelling adults in a French-speaking Swiss municipality since 2019 (Plattet & Spini, 2021). The Cause Commune project was initiated to better understand social problems and to identify intervention pathways in Chavannes-près-Renens, a municipality in western Switzerland, with attention to social cohesion and disparities in well-being among residents (Plattet & Spini, 2021).

As a community-based participatory research project, Cause Commune aims to engage and promote the competencies of residents to bring about changes in their community (Minkler & Wallerstein, 2008; Wallerstein & Duran, 2006) through constant participation of residents and municipal partners. In the Cause Commune project, members of the municipality help identify issues and research questions, provide responses, and help generate solutions, whereas researchers collect and analyze data, disseminate findings, and develop interventions based on suggestions from residents and in consultation with municipal partners.

The Cause Commune project was designed to involve the entire adult resident population of Chavannes-près-Renens. The first wave was conducted in 2019 and the second wave in 2021. At the time of this writing, data analysis for the third wave has not been finalized. In the baseline wave (2019), all 6,220 eligible adults from the municipal registry were contacted, and 1,401 adult residents aged 18 or older provided valid responses to the survey questionnaire (Plattet & Spini, 2021; Spini et al., 2021). About 54% were female; 39% aged 18 to 40, 39% aged 41 to 64, and 21% aged 65 or older; 10% of the sample obtained primary education, 51% secondary education, and 39% obtained tertiary education.

### Ethical Approval

The Cantonal Commission on Ethics in Human Research is a cantonal administrative body in Switzerland established by the Law on Human Research to ensure the protection of research participants and to assess the compliance of human research projects with ethical, legal, and scientific requirements. The Cantonal Commission on Ethics in Human Research (CER-VD) decided that the Cause Commune project did not fall within the scope of human research in Switzerland.

### Interactive Map: Engaging Inhabitants’ Situated Knowledge

To identify health-enhancing geosocial resources in the community, we developed a web-based interactive map by engaging local inhabitants’ situated knowledge of their living environment. This method facilitates the collection of both quantitative and qualitative data. In the first phase of this mapping exercise, a task group named “La santé dans tous ses états” (literally, health in all its dimensions) was recruited and organized by the municipal social cohesion service in October 2021 in Chavannes-près-Renens, as part of the Cause Commune project. The aim of this task group was to encourage inhabitants to reflect and deconstruct the material and social resources in the community that they perceived as health-enhancing. Inhabitants took part in sketch mapping by placing colored markers on a satellite map of the municipality to indicate locations for health resources. Participants then provided qualitative comments on what they perceived as health-enhancing or health-impeding venues, possible causes and consequences for such observations, and what challenges they faced in accessing these resources (Minkler & Wallerstein, 2008). The qualitative comments were initially captured on paper and then transcribed to the electronic database. To preserve confidentiality, all comments were fully anonymized. In the second stage of the mapping exercise, the places identified in the first stage were geo-coded, visualized in four spatial layers (one layer per health domain), and illustrated in a public interactive map using a quantum geographic information system (QGIS). In particular, the geographic coordinates corresponding to each place, together with the comments associated with them, were integrated into GIS layers, before all layers were spatially joined to satellite and transportation base maps with street names and landmarks (e.g., train stations and parks) to facilitate situational recognition. Finally, to present the geosocial information in a user-friendly manner, a web-based interactive map was generated using QGIS.

### Intersectional Matrix: Identifying Precise Social Locations

To identify precise social locations for targeted intervention on loneliness, we developed an intersectional matrix based on the reality that each person simultaneously belongs to multiple demographic categories (e.g., an older migrant woman with advanced education), utilizing quantitative data collected from the Cause Commune project. We measured loneliness with four items taken from the scale for overall, emotional, and social loneliness (De Jong Gierveld & Van Tilburg, 2010): “I miss having people around me”; “I often feel rejected”; “There are many people I can trust completely”; “There are enough people I feel close to.” This matrix represents the outcome of prior analyses using a multilevel modeling technique identifying contextual risk levels (Li & Spini, 2022) in the community where social groups with similar intersectional identities share common exposures to social processes, conceptually grounded in the intersectionality framework (Crenshaw, 1989; Merlo, 2018; Yuval-Davis, 2011). In essence, to capture the precise social location of individuals, we used four dimensions of social identity to construct the intersectional strata: age, gender, educational attainment, and nationality, and estimated random effects at the intersectional stratum level (Li & Spini, 2022). We used this method to generate precise estimates showing how the level of loneliness varied when the four multiplicative attributes (i.e., age × gender × education × nationality) were jointly conceptualized as social contexts that corresponded to people’s intersecting identities. This method enables the quantitative examination of intersectionality. In this paper, we focus on communicating these results to the public audience. For statistical and technical details, see Li and Spini (2022).

### Health Radar: Visualizing Multidimensional Longitudinal Comparisons

A relatively simple yet effective strategy to visualize multidomain comparisons over time is radar charts (Porter & Niksiar, 2018). Although radar charts are not frequently used in the social sciences, they offer a visual summary of multiple dimensions in a single graphic consisting of closed polygonal profiles of predetermined shape, size, and position (Dzemyda et al., 2013; Porter & Niksiar, 2018). We use radar charts to illustrate the contextual embeddedness of multidomain health in the community, emphasizing the social groups and relationships inherent in social quality, a multidimensional conceptualization and measurement of the well-being of societies as well as individuals embedded within them (van der Maesen & Walker, 2005). Prior research suggests four domains of social quality: socio-economic security, social cohesion, social inclusion, and social empowerment, based on the assumption that these domains influence health and well-being (van der Maesen & Walker, 2005). This makes use of the key advantage of radar charts—a centralized display of strengths and weaknesses in particular domains over time. Specifically, we plot seven domains of health and social quality, each of which represents a radial axis. The center of the polygon represents the value of zero, and the further toward the edge an indicator registers, the higher the quantity. The overall display thus enables both within- and between-domain comparisons, making it possible to identify domains of strength (higher value, more pronounced) and weaknesses (lower value, less pronounced). Following the same logic, we plotted two waves of the Cause Commune data (2019 and 2021) for a longitudinal comparison of the seven domains, to identify areas that have improved (expanding toward the edge) and weakened (regressing to the center) over time. The questions used to assess these seven domains of health and social quality are provided in the Supplemental Material.

## Results

**Figure 2.**
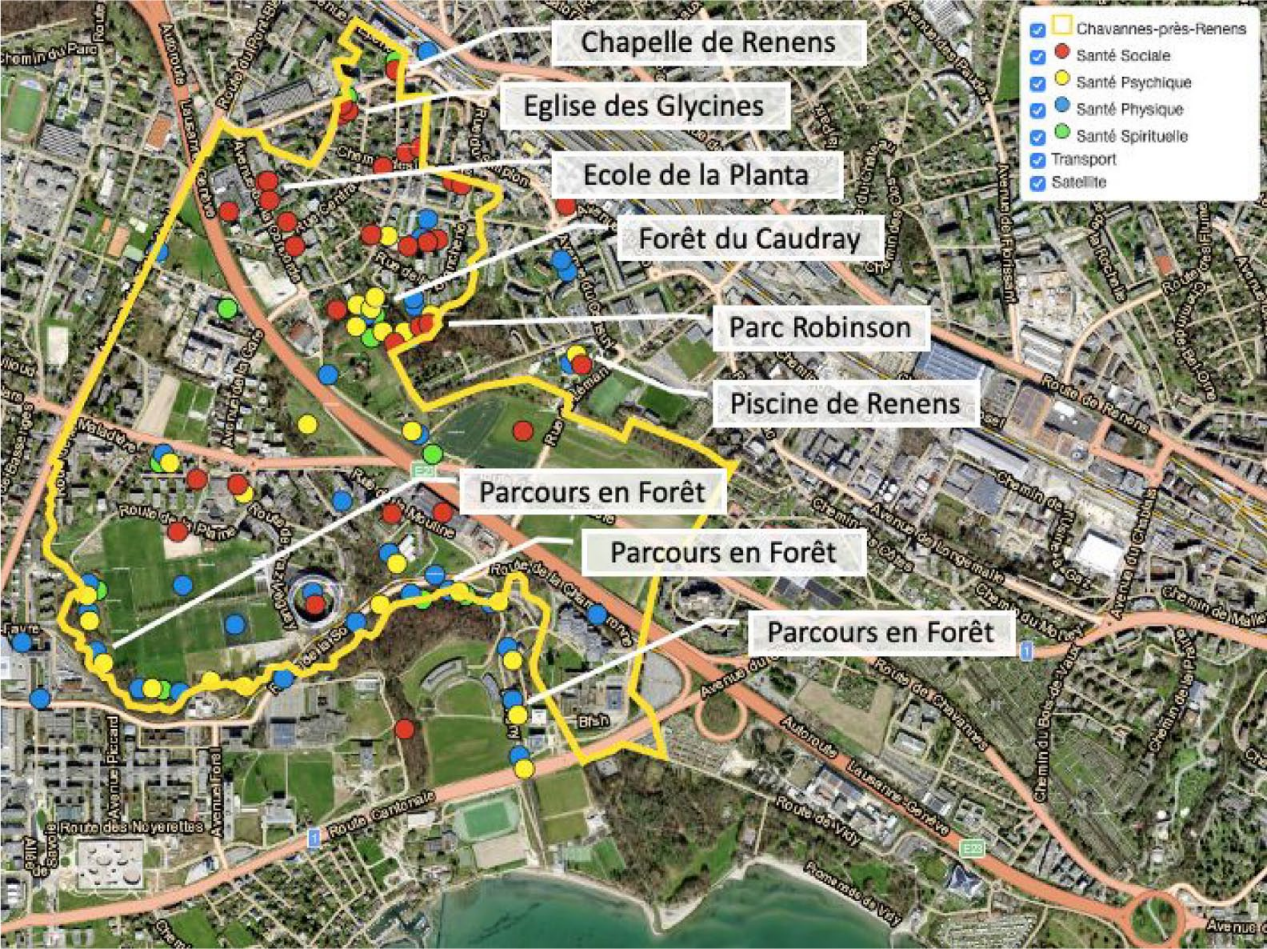
Interactive Map With Selected Health-Promoting Venues in the Community. *Source.* Li et al. (2022).

What places enhance health in the community? Through the interactive map, we identified 112 places that enhanced physical, mental, social, and spiritual health based on responses collected from the resident task group “La santé dans tous ses états.” First, the map revealed a clustering of health-enhancing places around existing public venues (Figure 2). Sites beneficial to social health (red markers) concentrated near “Ecole de la Planta,” a school in the community, while places that enhance physical health (blue markers) and mental health (yellow markers) formed clusters around existing recreational venues (e.g., Forêt du Caudray, Parc Robinson, Parcours en Forêt). Second, a handful of places simultaneously supported multiple domains of health, pointing to the multifunctionality of community spaces. For instance, while Parc Robinson provides green spaces for relaxation and physical health, it was also identified by local inhabitants to support spiritual health, social health, and mental health. Similarly, Piscine de Renens, a swimming pool and water sports facility, was perceived as simultaneously benefiting physical health, mental health, and social health. Third, social and material infrastructure both contribute to health and quality of life in the community. While material infrastructure including parks and swimming pools benefited physical and mental health, social infrastructure such as churches and chapels (e.g., Eglise des Glycines, Chapelle de Renens) also contributed to spiritual and social health, an essential part of overall quality of life.

**Figure 3.**
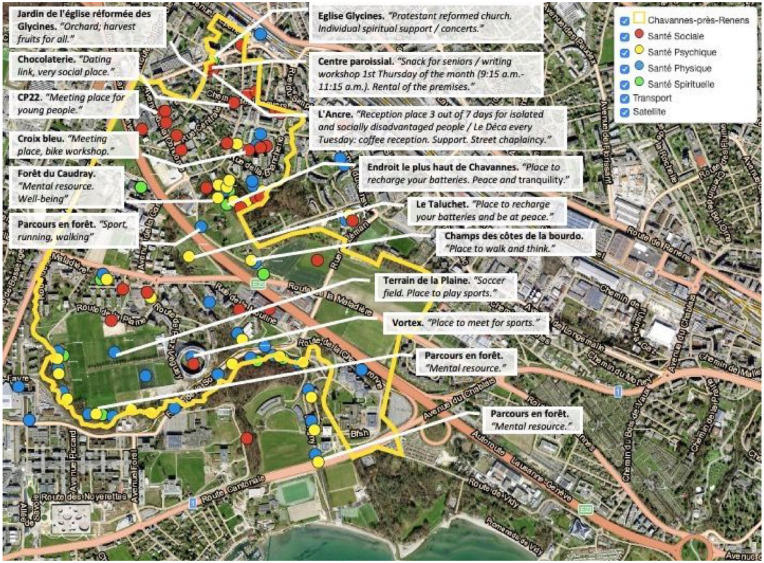
Interactive Map With Selected Qualitative Comments From the Inhabitants. *Source.* Authors’ work. *Note.* The names of the health sites are in bold font. Comments from the inhabitants are italicized and included in quotation marks. These comments were translated from French and were minimally edited for contextual clarity.

Why did participants identify these places as health-promoting? To understand their perception of a place, we paired respondents’ qualitative comments with the exact locations of health places on the interactive map to yield a better understanding of inhabitants’ perceptions of specific places (Figure 3). Example comments included:

“*Meeting place, bike workshop.*” (Social health)

“*Reception place 3 out of 7 days for isolated and socially disadvantaged people / Le Déca every Tuesday: coffee reception. Support. Street chaplaincy.”* (Social health)

*“Orchard; harvest fruits for all.”* (Social health)

*“Place to walk and think.”* (Mental health)

*“Place to recharge your batteries. Peace and tranquility.”* (Mental health)

*“Protestant reformed church. Individual spiritual support / concerts.”* (Spiritual health)

**Figure 4.**
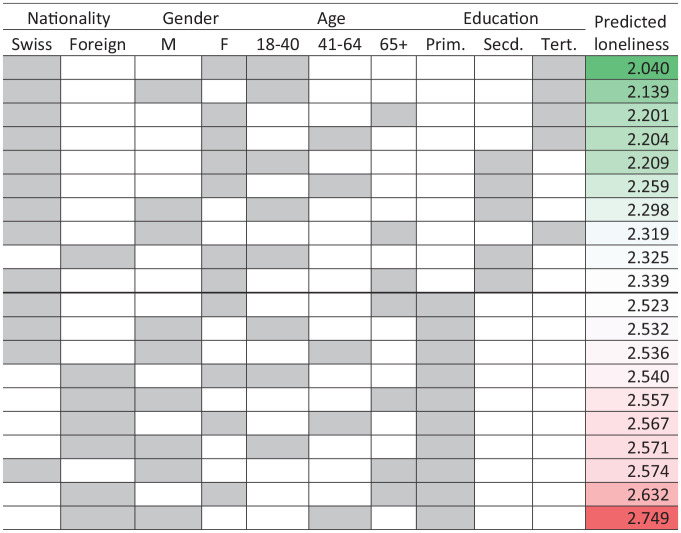
Predicted Stratum-Level Loneliness by Intersectional Attributes (10 Lowest and 10 Highest Strata). *Source.* Li and Spini (2022). *Note.* Loneliness: 1 = least lonely, 5 = most lonely. Shading: least at risk (top of the table) to most at risk (bottom of the table).

What social groups to target with interventions to address loneliness? Using the intersectional matrix, we show group-level predicted risk of loneliness based on inhabitants’ intersectional identities that cut across nationality, gender, age, and educational attainment (Figure 4). The groups with the highest levels of loneliness are mainly older migrant men without educational attainment beyond high school, as indicated by the strata toward the bottom of the matrix where the predicted risk of loneliness is highest. People with these intersectional identities are most at risk of loneliness and are logical intervention targets. By contrast, the groups with the lowest levels of loneliness are predominantly young Swiss women who have obtained secondary or tertiary education, as represented by the strata at the top of the matrix where the predicted risk of loneliness is lowest. Individuals sharing these intersectional social attributes are least at risk of loneliness. The intersectional matrix revealed the extent to which social identities of nationality, gender, age, and education shaped the risk of loneliness when these identities were treated as multiplicative rather than additive, as in real life each individual simultaneously belongs to multiple social categories, and no one is just an older woman or a younger migrant.

**Figure 5.**
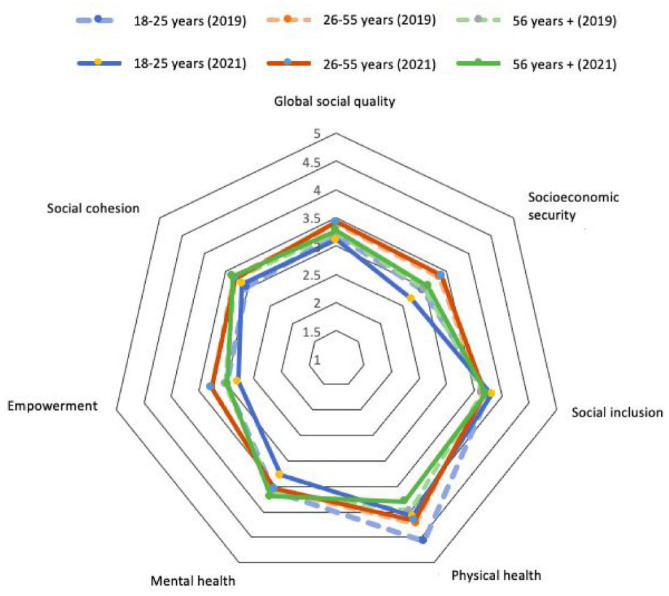
Radar Chart Comparing Multidomain Health and Social Quality by Age Group Over Time. *Source*. Spini et al. (2023).

Which age group fared worse in health from 2019 to 2021, and in what health domain? The radar chart (Figure 5) illustrates the multidomain comparison between age groups from 2019 to 2021 (the first and second waves of Cause Commune). The radar chart revealed that the youngest group (aged 18–25 years) suffered more during the period of the Covid-19 pandemic than other age groups, especially in terms of mental and physical health, but also socio-economic security and empowerment, as indicated by the axes of 2021 polygon for the age group of 18 to 25 (solid blue lines) regressing toward the center compared to that for 2019 (dashed blue lines). By contrast, the group aged 26 to 55 appeared to have weathered the pandemic period well relative to other age groups, as indicated by the 2021 polygon for the age group of 26 to 55 (solid red lines) overlapping the 2019 polygon for this age group (dashed red lines) almost completely, suggesting that most health and social quality domain for this age group remained unchanged, despite the multitude of economic, health, and social challenges during the pandemic period.

## Discussion

The objective of this study was to describe the use of geosocial communication tools for health promotion, in the context of multidomain health outcomes in a Swiss municipality. We drew insights from the conceptual framework of geosocial embeddedness to support the geosocial linkages between community and health, including the role of place, social groups, and social identities. Based on data collected from longitudinal surveys and participatory research engaging local inhabitants and their knowledge of the living environment, we demonstrated the use of (1) an interactive map that identified community spaces that promoted mental, physical, social, and spiritual health, (2) an intersectional matrix that delineated the precise intersectional social categories for risk of loneliness, and (3) a health radar chart that illustrated comparisons of health domains over time by specific social groups. Overall, these communication tools offered diagnoses of the most vulnerable social groups for tailored interventions and provided insight for policymakers to improve infrastructure to promote health in the municipality.

While limited prior research proposes similar geosocial tools for health communication, we highlight literature with relevant tools or mechanisms that promulgate community health information to support evidence-based policy and intervention development. Our findings are consistent with Zhou et al. (2016), who used a web-based participatory map to explore children’s perceptions of outdoor play places. Zhou and colleagues (2016) found that outdoor play spaces in Yantai, China clustered around existing public venues and that social interactions were among the reasons why inhabitants were motivated to utilize outdoor play spaces. Our findings also align with Termansen et al. (2023), which used community-based participatory approaches to promote health in a disadvantaged neighborhood in Copenhagen, Denmark. Termansen and colleagues (2023) considered the role of both the social and material dimensions of the living environment and found that open, flexible, and informal public spaces were central to community participation and social interaction, particularly among the most vulnerable population. Consistent with our research, Saary (2008) used radar charts to communicate multivariate health data and concluded that the health radar is a simple and effective strategy for visualizing multigroup comparisons of similarities and differences, particularly over multiple domains.

Our research contributes to the literature in several ways. First, the study is among the first to document health communication tools based on the framework of geosocial embeddedness. This framework highlights the interaction between social processes, social actors, and the role of places in the day-to-day activities of the community. Using an interactive map to identify health spaces in the community, we illustrated the synergistic power of the geosocial framework through the multifunctionality of physical and social places that promote health. Using an intersectional matrix to identify precise social categories for interventions to reduce loneliness, we demonstrated the contextual embeddedness of social identities and the potential to utilize simultaneous social identities for precise community diagnosis and interventions. Second, the study draws from community-based participatory research where members of the municipality help identify issues and research questions, provide responses, and help generate solutions while researchers collect and analyze data, disseminate findings, and develop interventions based on suggestions. This approach, when combined with the communication tools we have described, facilitates researchers to extract inhabitants’ situated knowledge of the community and return research outcomes to inhabitants and other stakeholders, with the goal of improving the living environment and quality of life for everyone. Third, the study combines qualitative and quantitative data to understand what policy and intervention mechanisms will improve health in the community. Qualitative data from the inhabitants shed light on the reasons why existing community spaces were health-enhancing and potential areas of improvement. Quantitative data offered insight into the variability of loneliness by intersectional identities as well as cross-group comparisons on multidomain health indicators over time.

This study has important implications for policy and practice. First, the interactive map revealed not only the clustering of health territories around existing public venues, but also the multifunctionality of these territories in enhancing social, mental, and physical health. Municipal investment in relevant public infrastructure would be beneficial in continuing to motivate activities and interactions, advance social capital, and promote health. Greater investment in the maintenance and upgrade of these territories would contribute to the quality of life in the municipality. For administrators, the feedback on difficulty accessing some health sites or issues of noise and pollution implies that priority attention is needed in maintaining these places. For urban planners, the map offers insights into inhabitants’ preferences and perceptions of community places. Future designs to enhance safety, accessibility, and mixed-use facilities would prove particularly beneficial. Second, the intersectional matrix communicated the link between the intersectional identity context and the risk of loneliness, providing information for sharpened outreach efforts to alleviate loneliness by targeting those who are most at risk. Interventions may include customized communications and targeted promotion of social support, taking into account the linguistic and cultural background of the most vulnerable population segments. Third, the health radar chart sheds light on how different age groups fared in health outcomes over time, offering information on health promotion efforts to concentrate resources and address mental and physical health issues among younger adults, for example, as they have fared the worst in these health domains during the pandemic. Together, these geosocial tools communicate important information on the linkage between both the material and social dimensions of the community and health, catered to a public audience including inhabitants, administrators, urban planners, and others.

A few limitations merit discussion. First, the study provided an illustration of three example communication tools that translate empirical research on aspects of community health, conceptually based on the framework of geosocial embeddedness. The list of tools is not exhaustive, as other communication mechanisms may equally convey the linkage between the multidimensional community life and health. In selecting these tools, we focused on the potential for translating empirical research and communicating primarily with the public audience, including inhabitants themselves as well as agencies of social cohesion or municipal planning and safety. Second, our selection of tools was limited to conveying the geosocial aspects of community life, with consideration to diagnosing both the social and material dimensions of the community, using surveys and participatory research data we have available in the community of Chavannes-près-Renens. Third, our focus was to describe and demonstrate the tools of communication without belaboring the technical aspects. Where applicable, references were given for relevant literature containing detailed technical explanations. Fourth, due to data limitations, we were not able to demonstrate longitudinally all aspects of community life. Where data permit, future research should expand these communication tools to show longitudinal patterns in other health outcomes, especially using the health radar chart. Future research should emphasize network embeddedness within the geosocial framework and envisage relevant tools of communication. In addition, future research should investigate intersectional social categories in relation to other domains of health and quality of life, including material deprivation, precarity, and aspects of mental and functional health.

## Conclusion

Underpinned by the geosocial framework on the interaction between social processes, social actors, and the role of places, this research establishes a set of graphical and cartographic tools that communicate research results to facilitate the development and implementation of social policies in the municipality. These innovative tools have been co-constructed with local inhabitants and the municipal agency of social cohesion to induce changes in the municipality, centering on health, social environment, and material spaces. This community-based participatory research approach treats the community as a living laboratory where researchers partner with residents to assess the interactions between the social and material environment, and how their functions may promote health for precise community diagnosis and targeted health promotion.

## Supplemental Material

sj-docx-1-heb-10.1177_10901981241294230 – Supplemental material for Geosocial Tools for Community Diagnosis and Health Promotion Supplemental material, sj-docx-1-heb-10.1177_10901981241294230 for Geosocial Tools for Community Diagnosis and Health Promotion by Yang Li, Dario Spini and Cecilia Delgado Villanueva in Health Education & Behavior
